# Histological and micro-computed tomographic observations after maxillary sinus augmentation with porous hydroxyapatite alloplasts: a clinical case series

**DOI:** 10.1186/s40064-016-1885-2

**Published:** 2016-03-02

**Authors:** Hidemi Nakata, Shinji Kuroda, Noriko Tachikawa, Emi Okada, Maho Akatsuka, Shohei Kasugai, Hisatomo Kondo

**Affiliations:** Department of Prosthodontics and Oral Implantology, Dental Hospital, Iwate Medical University, 19-1 Uchimaru, Morioka, Iwate Japan; Department Oral Implantology and Regenerative Dental Medicine, Graduate School of Medical and Dental Sciences, Tokyo Medical and Dental University, 1-5-45, Bunkyo-ku, Yushima, Tokyo, 113-8510 Japan

**Keywords:** Sinus lift, Alloplast, Bone regeneration, Micro-computed tomography, Porous hydroxyapatite

## Abstract

**Background:**

It is important to visualize the phenomenon which occurs in actual clinical cases to decide the timing of implant placement subsequently after sinus graft. Although several clinical cases of bone augmentation using xenograft have been evaluated, the number of those reports which have described bone remodeling by alloplasty are not sufficient.

**Findings:**

In the present report, to investigate bone remodeling histologically after maxillary sinus augmentation with porous hydroxyapatite alloplast, bone cores from the sinus floor of three female nonsmoking patients (aged 64–73 years) were collected 6 months after sinus lift surgery, embedded in methyl methacrylate resin, and prepared by conventional methods. Bone architecture and graft residues were evaluated by micro-computed tomography of the same specimens. Hematoxylin–eosin and Villanueva–Goldner staining revealed mature osteoblasts and multinucleated osteoclasts on the grafted sinus floor and surface of residual hydroxyapatite particles. The particulate interspace was partially filled with osteoid and calcified tissue and showed active vascularization.

**Conclusion:**

The results suggested that bone regeneration and angiogenesis within and between porous hydroxyapatite particles were sufficiently found after 6 month histologically in the grafted sinus floor.

**Electronic supplementary material:**

The online version of this article (doi:10.1186/s40064-016-1885-2) contains supplementary material, which is available to authorized users.

## Background

Porous hydroxyapatite is a major bone substitute (Yoshikawa et al. 2009; Yuasa et al. 2004). Its high pore rate (72–78 %) and large interpore distance markedly reduce its mechanical strength (Moller et al. 2014; Liu 1996). However, greater porosity facilitates intraparticulate cell migration from adjacent tissues (Phipps et al. 2012; Fassina et al. 2010). Bone conductivity of hydroxyapatite also induces bone formation in the particles (Campana et al. 2014). Hydroxyapatite particles can be used to maintain the lifted sinus space and promote mineralization and maturation of new bone; however histological and radiographic findings of bone regeneration clinically induced by porous hydroxyapatite alloplast have not been frequently reported (Kolerman et al. 2012). Difficulty in the surgical procedure can be a major reason why only a few number of this kind of works have been reported, wherein just a single drilling in the jaw with a trephine bur is allowed to retrieve the bone-with- HA sample, simultaneously preparing the direction and cavity diameter for the implant at once. And it is often that mishandling with the drill bit would not be recovered, resulting in missing of the primary stabilization of implants. Furthermore, it might not always be easy to obtain patients’ understanding of the unusual methodology of the implant surgery and eligible to proceed with it for benefit to patients.

In this study, bone cores from the sinus floor of three patients were collected 6 months after maxillary sinus augmentation with porous hydroxyapatite alloplast to investigate bone formation histologically and evaluate bone architecture and graft residues by micro-computed tomography (CT).

## Methods

All experiments were approved (approval number 884) by and followed the guidelines of Tokyo Medical and Dental University.

### Patients

**Fig. 1:**
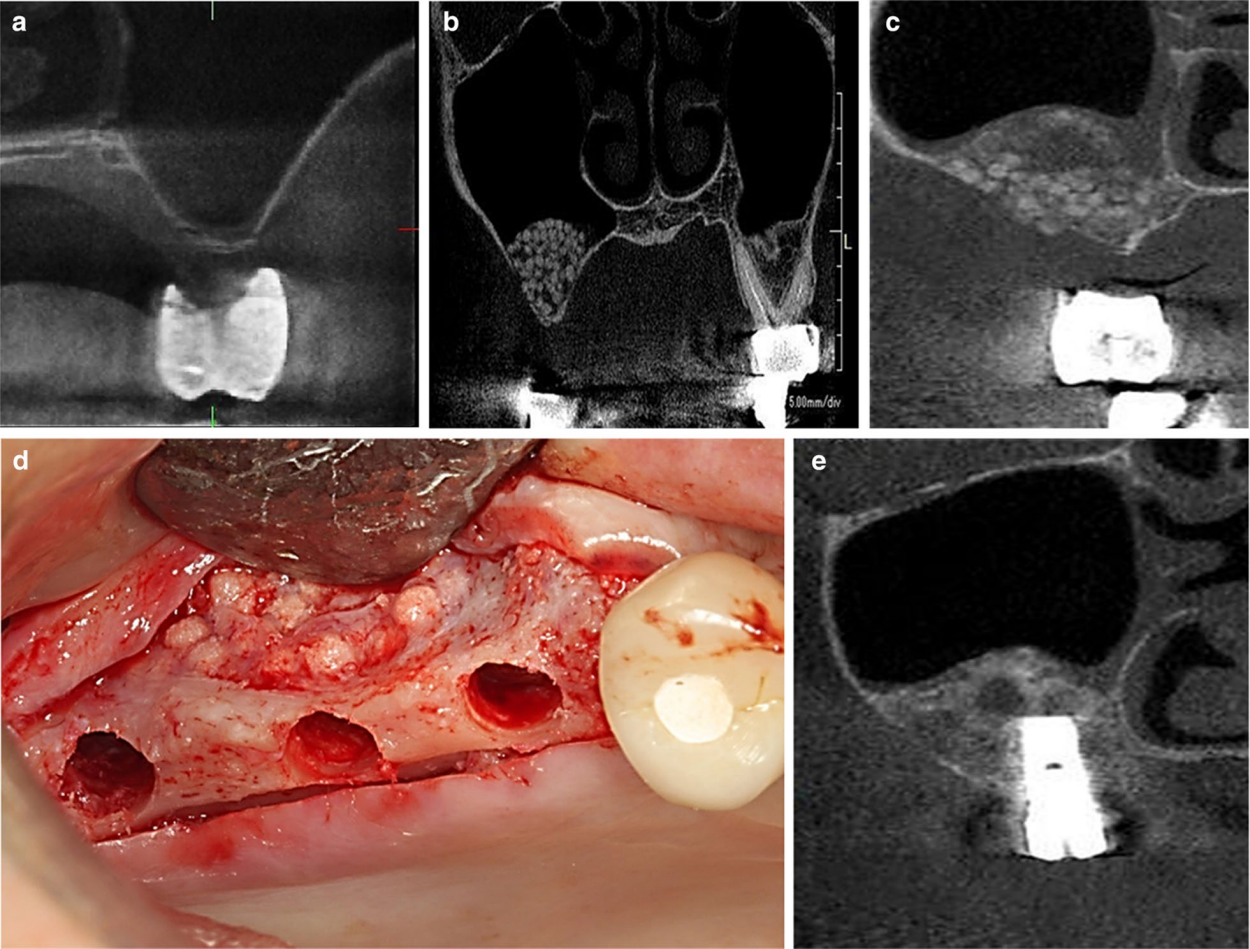
CT scan before sinus graft. The original bone thickness from the alveolar ridge to the sinus floor was approximately 1–2 mm in average (**a**). CT image after maxillary sinus augmentation (**b**). A postoperative image for estimating the dental implant placement site (**c**). Photograph obtained after harvesting the bone cores and preparation for dental implant placement (**d**). Hydroxyapatite particles are visible in the lateral window. The operated sinus floors did not shown signs of inflammation.* Scale*  1 mm

Three female patients (aged 64–73 years) with severe alveolar bone resorption originating from periodontal disease or trauma were enrolled in this study. None smoked or had systemic pathologies affecting the immune system and contraindications for surgery and sinus grafting. Preoperative CT revealed clear maxillary sinuses and bone height from the alveolar ridge to the sinus floor of 1–2 mm (Fig. 1a). All the patients gave written consent for bone removal during dental implant surgery and its analysis.

The patients underwent sinus lift surgery under sedation and local anesthesia from the lateral approach according to the protocol described by Boyne and James (1980). After sinus membrane elevation, porous hydroxyapatite particles (φ 1–2 mm, 1 g; NeoBone, Covalent Materials Corporation, Tokyo, Japan) were grafted onto the sinus floor from the lateral window (Fig. 1b).

### Collection of bone core and implant placement

Six months after sinus lift surgery, a full-thickness flap was raised and the sites for dental implant placement were marked with a φ 2-mm round bur to a depth of approximately 2 mm to remove the residual cortical bone in collected bone core. Bone cores were removed until approximately 6 mm vertically from the alveolar ridge by using a trephine bur (3-mm outer diameter), and dental implants (Astratech Implant System [4.0ST × 9 mm], Dentsply Implants, Mannheim, Germany, Brånemark System MkIII TiU [φ 3.75 × 10 mm] or NobelReplace Tapered [φ 4.3 × 10 mm], Nobel Biocare, Göthenburg, Sweden) were placed into the sockets. The stabilization torque during placement was around 15–20 Ncm. All the implants were submerged with 0-mm-high cover screws.

### Specimen preparation

The bone cores were immediately fixed in 10 % paraformaldehyde (pH 7.3), dehydrated in 70–99.5 % ethanol and acetone for 1–2 days at each stage, exposed to a 1:1 ratio of acetone and methyl methacrylate (MMA) monomer for 3 days, further treated with MMA monomer for 10 days, embedded in MMA resin, and allowed to harden for 12 days. They were then cut by using a precision sectioning saw (IsoMet-1000, Buehler, Lake Bluff, IL) and sliced into 6-μm-thick sections (nondecalcified) with a rotary microtome (Leica RM2255, Leica Biosystems, Wetzlar, Germany).

### Histological analysis

Sections were deresinated with xylene for 2 h at 60 °C. For Villanueva–Goldner staining, deresinated sections were stained with iron hematoxylin for 20 min, treated with 1 % hydrochloric acid–ethanol solution, stained with Ponceau fuchsin for 120 min, treated with 1 % acetic acid, and stained with phosphotungstic acid–phosphomolybdic acid solution. They were then stained with naphthol green solution for 30 min and washed with 70–99.5 % ethanol. For hematoxylin–eosin staining, deresinated sections were stained with iron hematoxylin for 20 min, treated with 1 % hydrochloric acid–ethanol solution, stained with eosin solution, and washed with 99.5 % ethanol. After treatment with xylene, all the stained sections were enclosed and observed with a light microscope (Olympus, Tokyo, Japan).

### Micro-CT analysis

The bone cores were also scanned with a microfocus X-ray CT system (Scan Xmate-L090, Comscantecno Co., Ltd., Yokohama, Japan). The X-ray source was set at 80 kV and 100 μA, and the samples were rotated 360°. Image resolution was fixed at a pixel size of 11.587 μm. The magnification was 8.630, and slice thickness was 11.587 μm. Three-dimensional measurements and structural analyses were performed with custom software (TRI/3D-Bon, Ratoc System Engineering, Kanagawa, Japan). The images were binarized with a threshold range between 1 and 255 (gray values). To distinguish grafted and native bone from the background, a suitable threshold range is required for grafted bone: the minimum value was 31 and gradation range was 224 for native bone with hydroxyapatite particles, and the minimum value was 123 and gradation range was 132 for hydroxyapatite particles alone. The parameters included percentage of bone volume (BV), bone surface to bone volume (BS/BV), bone volume-to-tissue volume (BV/TV), trabecular thickness (Tb.Th), trabecular number (Tb.N), trabecular separation (Tb.Sp), trabecular spacing (Tb.Spac), and fractal dimension (D) were analyzed. BV/TV is the percentage of BV relative to TV within the bone core. Tb.Th represents the mean thickness of individual trabeculae, and Tb.Sp shows the space between trabeculae within a sample. Tb.Pf was developed by Hahn et al. (Browaeys et al. 2007): lower Tb.Pf signifies better connected trabecular lattices, while higher Tb.Pf indicates more disconnected trabecular structure.

## Results

### Surgical outcomes

The postoperative course was uneventful, and the amount of hard tissue was sufficient for dental implant placement evaluated by preoperative CT (Fig. 1c). The stability of the bone substitute was favorable (Fig. 1d). The implants showed around 20–25 Ncm of torque for placement, and were stabilized enough to resist the movement which prevent osseointegration of the dental implants during healing periods (Fig. 1e).

### Histological findings

**Fig. 2:**
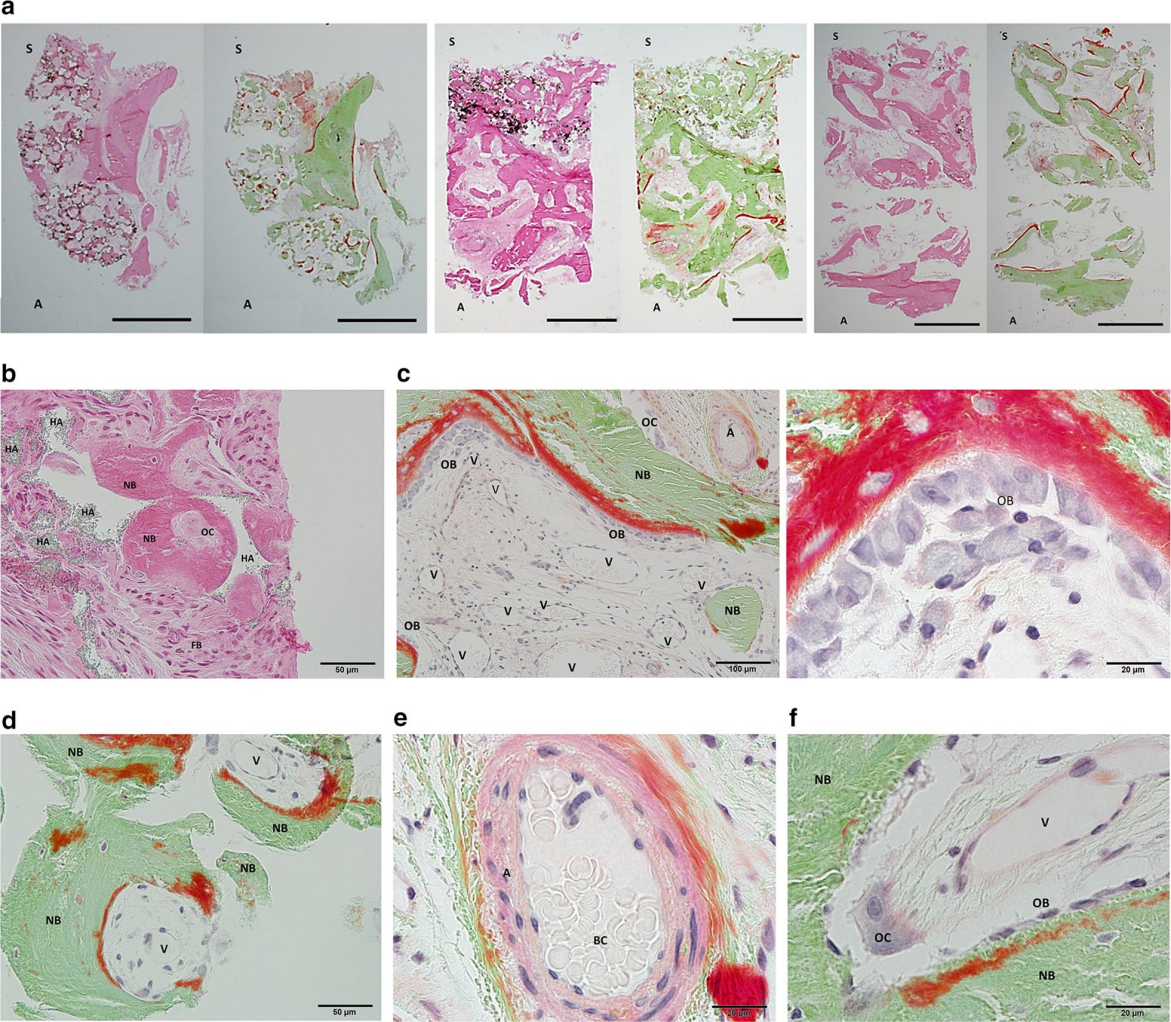
Low-magnification (×40) images of stained vertical sections of whole bone blocks. (**a** 1–3; *S* Snus floor. *A* Alveolar ridge). The *dark brown* coloration indicates residual hydroxyapatite in sections stained with hematoxylin–eosin (*left*). Bone and osteoid are indicated by *green* and *red*, respectively, in sections stained with Villanueva–Goldner trichrome (*right*). **b** has been shown high-magnification images of **a**-2. Residual hydroxyapatite (HA), migration of numerous fibroblasts (FB), and bone formation (NB) have been shown (×200; hematoxylin–eosin stain). An osteoclast (OC) is visible in bone formed in the pores of hydroxyapatite particles. Active vascularization (*V* vein, *A* Artery) and activated mature osteoblasts (OB) showing polygonal shape and numerous secretory vesicles on the osteoid surface (*red*) can be seen in **c** (1: ×100, 2: ×1000; Villanueva–Goldner stain). High magnification of **a**-2 indicated in **d**: Globular bone formation (NB) in the pores of hydroxyapatite particles has been shown (×200; Villanueva–Goldner stain). Small arteries showing blood cells were observed (**e**: ×1000; Villanueva–Goldner stain). A multinuclear large osteoclast was observed with osteoblasts and vascularization on the surface of new bone (**f**: ×1000; Villanueva–Goldner stain)

Each bone core represents different histological view (Fig. 2a, 1–3). The lower side indicates the alveolar ridge in Fig. 2a 1–3. Numerous fibroblasts (FB) which supposed to differentiate into osteoblasts migrated into the surface of pores of hydroxyapatite particles, and newly formed bone (NB) was observed with mature multinucleated osteoclast (OC) at the interspace of hydroxyapatite residues (HA) (Fig. 2b). Activated mature osteoblasts (OB) with secretory vesicles were located at the surface of osteoid (red) which engrails the newly formed bone (green) (Fig. 2c-1: low magnification, Fig. 2c-2: high magnification). Bone formation has been taken place along the interface of the hydroxyapatite pores (Fig. 2d). New blood vessels (V: vein, A: artery) were also found in the particles and surrounding areas (Fig. 2d, e). Osteoclasts (OC) that found at the interface of new bone (NB; green) and hydroxyapatite (blank) may absorb the surface of particles (Fig. 2f).

### Micro-CT findings

**Fig. 3:**
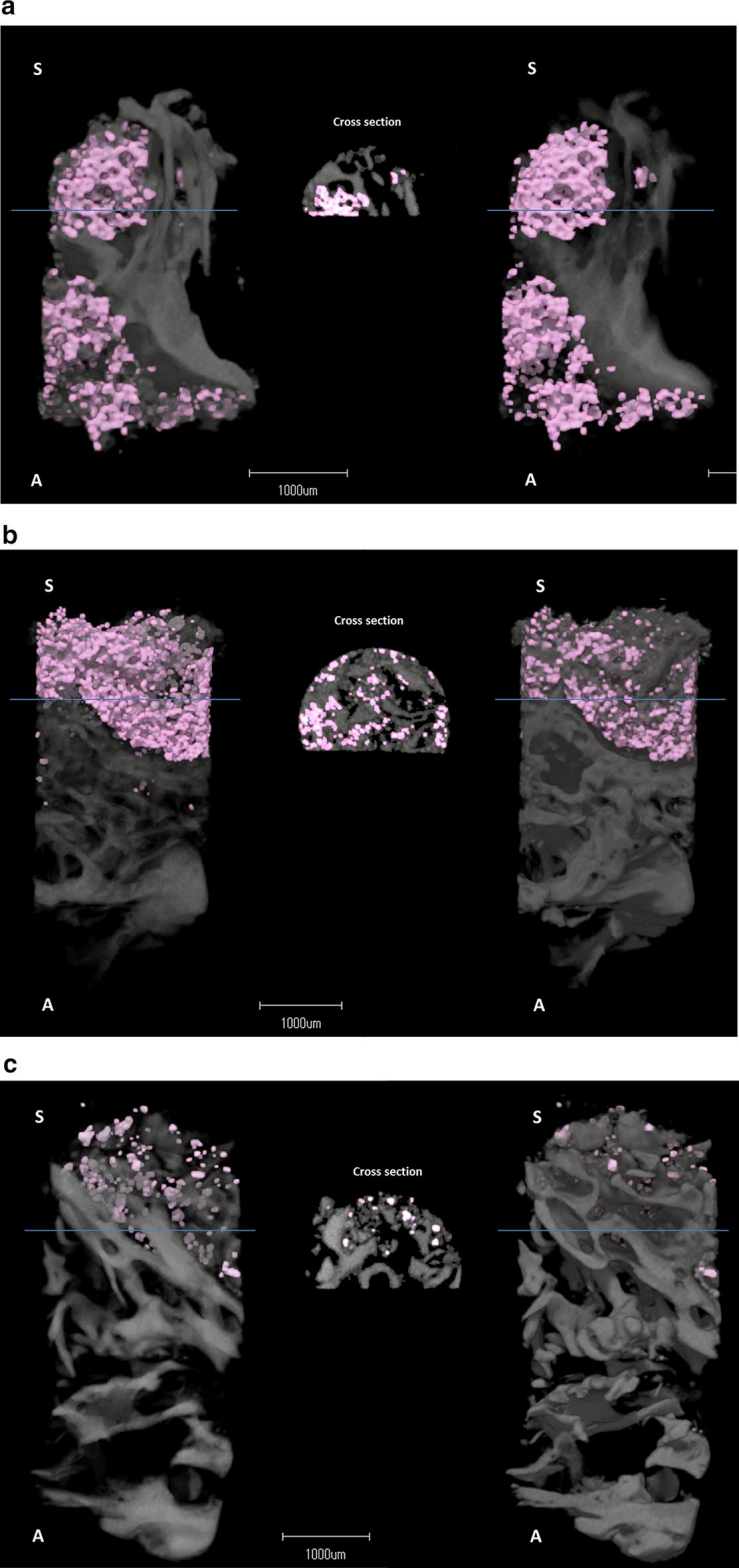
Micro-CT scans of trabecular bone. Vertical 3-dimensional images and cross-sectional 2-dimentional images at the level of the *blue* line (**a**–**c**; *S* Snus floor. *A* Alveolar ridge) and movie of horizontal rotation (Additional files 1, 2, 3) of the bone core are shown. A fixed threshold range (123–132) was applied to segment the grafted bone from the background. New bone is observable in the grafted sites. Residual hydroxyapatite is indicated in* purple*; its volume was not equal in the samples

The architecture of the bone cores was different between each other and the volume of residual hydroxyapatite (purple) also varied among the patients (Fig. 3a–c, Additional files 1, 2, 3). The lower side indicates the alveolar ridge in Fig. 3a–c. One case shows abundant residues of hydroxyapatite from the alveolar ridge to the bottom of the bone core (Fig. 3a), whereas, hydroxyapatite residues were located at the distal region from the alveolar ridge in two cases (Fig. 3b, c). There can be seen residual bone in the right side of the bone core, and also observed outgrowth of the bone structure from the original bone into the hydroxyapatite particulate interspace (Fig. 3a). New bone and osteoid were appeared as a blanched area in all the patients (Fig. 3a–c, cross sections; middle of the figures). The radiographic data are summarized in Table 1. The mean BV/TV was approximately 30 %, and mean hydroxyapatite residue was 1.2 % (Table 1). The residual original bone area of Fig. 3a was avoided for the calculation and analysis in this study to evaluate only the grafted area.

**Table 1:** Summary of the micro-CT analysis for native bone (B) + hydroxyapatite (HA) and HA residue

	“TV”	“BV”	“BS”	“BS/BV”	“BV/TV”	“Tb.Th”	“Tb.N”	“Tb.Sp”	“Tb.Spac”	“D”
	[mm^3^]	[mm^3^]	[mm^2^]	[1/mm]	[%]	[urn]	[1/mm]	[urn]	[urn]	
B + HA										
Subject 1	6.439	2.063	45.788	22.190	32.0	90.130	3.556	191.103	281.232	2.254
Subject 2	12.831	4.584	120.607	26.313	35.7	76.008	4.700	136.772	212.780	2.358
Subject 3	13.563	2.796	74.651	26.698	20.6	74.912	2.752	288.453	363.365	2.215
HA										
Subject 1	6.439	0.132	9.396	71.169	2.1	28.102	0.730	1342.363	1370.465	2.033
Subject 2	12.831	0.197	18.987	96.247	1.5	20.780	0.740	1330.835	1351.615	1.888
Subject 3	13.563	0.015	1.645	107.744	0.1	18.562	0.061	16,474.093	16,492.655	1.474

*TV* tissue volume, *BV* bone volume, *BS* bone surface, *Tb.Th* trabecular thickness, *Tb.N* trabecular number, *Tb.Sp* trabecular separation, *Tb.Spac* trabecular spacing, *D* fractal dimension

## Discussion

In the present study, bone formation and vascularization were observed 6 months after sinus augmentation with porous hydroxyapatite alloplast. These results confirmed the evidence that 6 month considered as a standard healing period subsequently to sinus grafting would be sufficient for preparation of implant placement (Tatum 1986). Kurabuda, et al. described differences of newly formed bone/residual material ratio between 3 kinds of graft materials; demineralized freeze-dried bone powder (DFBP), deproteinized bovine bone (DBBG), and porous hydroxyapatite (PHA) at 6 month after sinus graft, and their results showed that the lowest rate of newly formed bone was observed in patients who received PHA (Karabuda et al. 2001). And they concluded prolonged healing periods may be required for the cases using PHA. Reducing time of procedure has been considered beneficial for patients; however, further studies for verification of each material at each time point will be necessary to confirm the protocol. Since the preoperative bone height, systemic conditions and other related parameters are not constant in clinical cases, densities of calcified tissue and resisual hydroxyapatite will be different even with the same materials and protocols. Therefore, it can be complicating to compare the clinical outcomes directly between different materials. Currently, hydroxyapatite xenoglaft and alloplast are common bone substitutes for alveolar augmentation clinically (Kolerman et al. 2012; Boyne 1980; Chaves et al. 2012). Although there has been literatures describing results of using xenograft (Cassetta et al. 2014; De Souza Nunes 2011; Lambert et al. 2013; de Vicente et al. 2010; Carlo Mangano et al. 2006; Orsini et al. 2005; Stevens et al. 2005; Browaeys et al. 2007), only some reports have been published about clinical investigation for alloplast (Kolerman et al. 2012; Minamiguchi et al. 2008). In this study, sinus augmentation with hydroxyapatite alloplast was performed and evaluated.

Micro-CT is useful for evaluating bone parameters but does not identify new bone or bone remodeling (Huang et al. 2013; Kuhl et al. 2013). In contrast, histology can reveal cell morphology, new bone or even osteoid (Proussaefs et al. 2003; Ewers et al. 2004; Groeneveld et al. 1999); however, it does not permit images of the three-dimensional architecture. In this study, analyses by both histology and micro-CT enabled clarified bone remodeling in the same sections from diverse perspectives. The results obtained from micro-CT revealed the BV/TV reached only around 30 % and hydroxyapatite residue was 1.2 % in average, these alone could not optimize osseointegration of the implants but, from histology, could explain the active bone remodeling and bone formation in the grafted region such as mature osteoclasts, activated thickened osteoblasts with numerous secretory vesicles, and extensive vascularization indicated continuous active bone remodeling.

Although the drilling depth by trephine burs was around 6 mm underneath the 2 mm thick cortical bone, the height of harvested columnar bone samples was approximately 4–5 mm. Therefore, bone formation at the interface of the sinus membrane and grafted bone was not evaluated in this study. However, this result might indicate the new bone generated from the residual bone such as alveolar ridge, septal wall and maxillary tuberosity due to location of the hydroxyapatite residues and the blanching morphology of the newly formed bone from this original bone. However, there is a repot of possibility of bone formation along the sinus membrane(Sohn et al. 2011), thus, further investigations are required to confirm the mechanisms of bone formation in the grafted sinus.

## Conclusion

Histological and micro-CT findings in bone cores at estimated implant placing sites indicate bone regeneration within and between porous hydroxyapatite allopllast 6 month after maxillary sinus augmentation.

## Additional files

10.1186/s40064-016-1885-2 Horizontal rotation movie corresponding to Fig. 3a.
10.1186/s40064-016-1885-2 Horizontal rotation movie corresponding to Fig. 3b.
10.1186/s40064-016-1885-2 Horizontal rotation movie corresponding to Fig. 3c.

## Abbreviations

CT: computed tomography
TV: tissue volume
BV: bone volume
BS: bone surface
Tb.Th: trabecular thickness
Tb.N: trabecular number
Tb.Sp: trabecular separation
Tb.Spac: trabecular spacing
D: fractal dimension
V: vein
A: artery
NB: new bone
OB: osteoblast
OC: osteoclast
HA: hydroxyapatite
FB: fibroblast
BC: blood cell
